# Nursing workload evaluation in ICU using the TISS-28 scale in cardiac surgery patients

**DOI:** 10.1186/2197-425X-3-S1-A718

**Published:** 2015-10-01

**Authors:** O Didenko, V Isajevas, D Gineityte, V Vicka

**Affiliations:** Vilnius University Hospital Santariskiu Clinics, Vilnius, Lithuania; Vilnius University, Faculty of Medicine, Vilnius, Lithuania

## Introduction

The nursing workload is an important factor determining the outcomes of patients admitted to the ICU after cardiac surgery. An elevated nursing workload increases the amount of errors and the rate of postoperative complications.

## Objectives

To evaluate the nursing workload in ICU and assess the risk factors of increased nursing workload.

## Methods

The study was conducted in tertiary hospital reference center and included patients undergoing cardiac surgery. The nursing workload was assessed using the TISS-28 scale. Euroscore II predicted operative risk, type of the surgery and age of the patient were investigated as a possible risk factors of increased nursing load. Furthermore, an analysis of TISS-28 components was carried out to determine the nursing activities which accounted for an increased nursing workload. Appropriate statistical tests were used in statistical analysis.

## Results

1146 nursing workload estimations were gathered. The mean TISS-28 value for one patient was 29,99 ± 9,34 points. Percentage distribution of the mean TISS-28 score was as follows: Basic activities (BA) 39,64%, Cardiovascular support (CS) 27,54%, Ventilation support (VS) 11,97%, Renal support (RS) 13,1%, Metabolic support (MS) 4,6% Specific intervention (SI) 3,01%, Neurologic support (NS) 0,02%. The mean nursing workload was 56,31 ± 21,17 points. The nurses were divided into two groups: normal workload (TISS-28 value < 45) consisting 32,6% (n=198) and not (TISS-28 value ≥45) consisting 67,4% (n=409). Significant differences were found in points scored for BA, CS, SI, RS and MS between these two groups. Furthermore, preoperative factors of increased nursing workload were analyzed. To do so, the group was randomized using an Euroscore II cut-off point of 10. Coronary artery bypass surgery (38 (17,9%) vs 4 (6,7%) p=0,041), other causes of ICU admission (26 (15,4%) vs 16 (26,7%) p=0,006) and Euroscore II>5 (97 (45,8%) vs 17 (28,3%) p=0,016) were associated with increased nursing workload, which unfolded as risk factors in regression analysis: OR=2,13 CI95% 1,14-3,98 p=0,017, OR=0,38 CI95% 0,19-0,78 p=0,008 and OR=3,06 CI95% 1,05-8,94 p=0,041, respectively.

## Conclusions

The nursing workload exceeds recommended standards in ICU patients after cardiac surgery. Euroscore II predicted operative risk and type of surgery elevate the risk of increased nursing workload. We propose these factors to be accounted for in rational resource allocation.

